# CT Anatomical and Morphometric Study of the Red Fox (*Vulpes Vulpes*): Cervical Vertebrae

**DOI:** 10.1155/vmi/7730180

**Published:** 2025-12-09

**Authors:** Yasin Valizadeh, Mohsen Abbasi, Omid Zehtabvar, Amir Zakian, Ali Reza Vajhi, Ferdos Fekri

**Affiliations:** ^1^ Department of Clinical Sciences, Faculty of Veterinary Medicine, Lorestan University, Khorramabad, Iran, lu.ac.ir; ^2^ Department of Basic Science, Faculty of Veterinary Medicine, Lorestan University, Khorramabad, Iran, lu.ac.ir; ^3^ Department of Basic Science, Faculty of Veterinary Medicine, Anatomy Sector, University of Tehran, Tehran, Iran, ut.ac.ir; ^4^ Department of Surgery and Radiology, Faculty of Veterinary Medicine, University of Tehran, Tehran, Iran, ut.ac.ir

**Keywords:** anatomy, cervical vertebrae, CT anatomy, CT scan, red fox

## Abstract

It is necessary to produce basic anatomical information for clinical examinations and necessary surgeries owing to the presence of the red fox in the wild and the health risks for these animals. In addition to being important in diagnosing animal injuries, imaging techniques provide the usual anatomical view of different body structures used in many studies. This study investigated the typical morphological and morphometric characteristics of normal, immature, and healthy male fox cervical vertebrae using a CT scan. A CT scanner with two detectors was used in the study. Several parameters were measured in five normal immature male and healthy foxes, and the results were evaluated. Some parameters, including vertebral body height (VBH) and vertebral body length (VBL), did not show any significant difference (*p* > 0.05) in the cervical site, but some parameters, including spinous process height (SPH), transverse process length (TPL), and transverse process width (TPW), had significant differences (*p* < 0.05) in the cervical site. VBH had a constant measure from the second cervical vertebra to the seventh vertebra. The value of TPL varied from the first to the seventh cervical vertebra, and the highest measure was observed in the first vertebra. This study presents a complete and precise description and morphometric evaluation of cervical vertebrae in immature male red foxes using a CT scan. No specimen was killed, and anatomical studies were conducted through a CT scan technique as an essential feature of this study.

## 1. Introduction

Red fox belongs to the *Chordata* phylum, the class *Mammalia*, the order *Carnivora*, and the family Canidae [1]. The red fox (*Vulpes vulpes*), also known as the common fox, has the broadest geographical range compared to other members of the carnivores [2]. This species can be found in most regions of Iran, except for the dense forests of the north, and its color is reddish brown. It is a valuable species of Iran’s wildlife, and a wide range of biological information must be collected to diagnose and treat various diseases to protect it.

The number of cervical vertebrae in carnivores, like most mammals, is 7. The first cervical vertebra is called the atlas, and the second cervical vertebra is called the axis. The third, fourth, and fifth cervical vertebrae are known as the typical vertebrae. The sixth and seventh cervical vertebrae are numbered [3].

The cervical vertebrae are the critical parts of the mammals’ skeleton, and studying their structure is extremely important. Given the importance of wild species, it is better to employ methods for anatomical studies that can be used on live animals, which ensure the animal remains alive and unharmed after the study. Different diagnostic imaging techniques, including a computed tomography (CT) scan, can be used for anatomical studies and investigating the internal structures of animals as noninvasive, precise, and fast methods. On the contrary, even though older methods, such as necropsy, are available and affordable, they are invasive methods unsuitable for investigating important wildlife species. In addition to indicating the actual position of the structures, a CT scan also enables a general anatomical view from different angles. Therefore, it is a suitable tool for interpreting vertebral column injuries, diagnosing complications and skeletal disorders, and anatomical studies [4].

Researchers have conducted various studies on the structure of the vertebral column in different animals, some of which are reviewed in this study. Sheng et al. examined the anatomical structures of the vertebral column in large animals and compared them with the vertebral column in humans. In this study, the animals included sheep, calf, deer, monkey, and pig. Significant differences were observed between humans and large animals regarding vertebral column anatomy. Monkeys and humans had more similarities in the cervical vertebral columns than other animals. The mean pedicle widths were similar between animals and humans, except for the thoracic vertebrae of sheep. The vertebral column was wider in humans than in animals, but the mean vertebral body height (VBH) was shorter than in all animals [5].

Penning et al. compared the level of cervical vertebrae movement in dogs and humans. In this study, it was reported that the level of movement in the cervical vertebrae of dogs was higher than in humans, and it depended to a large extent on the level of flexion and extension of the atlanto‐occipital joint. The flexion and extension patterns in dogs’ cranial and caudal cervical vertebrae were similar to humans [6].

Gilad et al. studied the geometrical sections of the human vertebral column in the longitudinal section. In this study, they used healthy people aged 20–38 years with an average height of 174 cm and examined the radiographs of 141 cervical vertebrae and 157 lumbar vertebrae under the same conditions and a standard procedure. The radiographic findings were as follows: The atlas vertebra is covered by the skull, which makes its geometrical identification and measurement impossible. Furthermore, the thoracic vertebrae were not evaluated as the longitudinal section of the ribs covered the thoracic vertebrae. In some cases, approximately 10% of the axis vertebrae could not be adequately detected, and the clavicle, humerus, and ribs covered approximately 10% of the C7 vertebrae. This study reported that the average vertebral body width increased from the axis to the C7 vertebra. The average VBH in the axis was bigger than that of other cervical vertebrae, while the other vertebrae had almost the same height [7]. In this study, the humerus bone began from the C4 vertebra. Furthermore, the forelimbs, including the scapula and humerus, were removed in the three‐dimensional (3D) reconstruction to examine the fourth to seventh vertebrae better.

This study was conducted due to the absence of accurate studies on the anatomical and morphometric structures of cervical vertebrae and a normal CT scan image of this site in red foxes. The study aimed to investigate CT anatomy and morphometric characteristics of cervical vertebrae.

## 2. Material and Methods

### 2.1. Animals

In this research, five immature and apparently healthy male red foxes (*Vulpes vulpes*) with the mean weight of 2.5 ± 0.05 kg were studied. All of them were males and were kept in the reproduction and rehabilitation center of Pardisan Park in Tehran under the same management and feeding conditions.

To prepare CT scan images after physical restraint, the outer covering of the desired section was first shaved from the paw of each animal, and after disinfection with alcohol, the catheter was fixed in the cephalic vein of each fox. Then, each animal received dexmedetomidine intravenously (at a dose of 20 μg/kg, Exir Pharmaceutical Company, Iran), together with ketamine (at a dose of 4 mg/kg, Bremer Pharma Company, Germany) to perform the CT scan under general anesthesia. The foxes were then transferred to the Veterinary Teaching Hospital (VTH), University of Tehran Diagnostic Imaging Center. After the completion of the imaging until the recovery from anesthesia, all foxes received normal saline 0.9% solution at a dose of 10 mL/kg/hour as the intravenous infusion. During the CT scan and recovery, the foxes were kept at constant room temperature (23°C–25°C) [8].

### 2.2. CT Scan Examination and Morphometric Evaluation

It should be mentioned that for bone studies, the CT scan is a better choice than radiography. It has some advantages over radiography, such as image magnification and the fact that the image can only be taken from certain angles, making CT scans a better choice for morphometric studies [9].

The animals were scanned under general anesthesia in ventral recumbency via a helical scanner (Somatom Spirit Series II CT scanner, Siemens, Berlin, Germany). CT scans were taken as transverse and perpendicular to the vertebral column from the cervical vertebral column. The technical factors of radiation in the CT scan method were as follows: rotation time: 1s, slice thickness: 1 mm, reconstruction interval: 2 mm, pitch: 1, X‐ray tube potential: 130 kV, and X‐ray tube current: 94–108 mA. The scan of each animal was recorded and evaluated by two independent board‐certified radiologists using “Radiant DICOM viewer” software. First, obvious anatomical structures were detected and marked in transverse and 3D reconstruction. Then, the length, width, and height of the spinous process; transverse process; and vertebral body were measured manually in duplicate using software ruler tools, and the mean of each measurement was recorded.

The anatomical parameters of a morphometric study that was evaluated in this study are listed in Table 1; however, the following points are worth mentioning:

1. Like other carnivores, the red fox does not have spinous process in the atlas vertebra; thus, the spinous process height (SPH) parameter was not included for the atlas vertebra.
2. Due to the existence of wings in the atlas vertebra, instead of the transverse process, the transverse process length (TPL) parameter is replaced with WPL, and also, the transverse process width (TPW) parameter was replaced with WPW.
3. The vertebral body length (VBL) parameter was measured in the sagittal view, and the rest of the parameters were measured in the transverse view.

**Table 1:** Anatomical parameters.

Parameter	Abbreviation	Description	Visual description in the red fox cervical vertebrae
Vertebral body height	VBH	Distance from the base of the vertebrae to the vertebral canal in transverse view; the maximum distance was measured	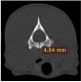
Spinous process height	SPH	Distance from the base to the apex of the spinal process; the maximum distance was measured	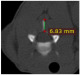
Transverse process length	TPL	Distance between the base of the transverse process and the extremity of the process in transverse view	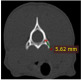
	WPL(C1)	Distance between the base of the wing process and the extremity of the process in transverse view	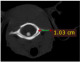
Transverse process width	TPW	Distance between the end of the right and left transverse processes in the transverse view; maximum distance was measured	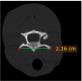
	WPW(C1)	Distance between the end of the right‐ and left‐wing processes in the transverse view; maximum distance was measured	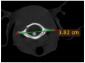
Vertebral body length	VBL	The length of the vertebral body in the midsagittal view; the maximum distance was measured	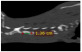

Finally, raw data were imputed into Excel software, and after visual observation to check the distribution of data, a one‐sample Kolmogorov–Smirnov test was utilized. Normally distributed data of morphometric measurements of different vertebrae were evaluated using a paired‐sample *t*‐test. Statistical analysis was performed via MedCalc statistical software (Version 20.104, MedCalc Software Ltd, Ostend, Belgium) at “*p* value” of < 0.05. It should be noted that morphometric measurements were performed in triplicate and by the same individual.

## 3. Results

Red fox cervical vertebrae were studied in this section. The 3D reconstructions were made to show the exact positions of these vertebrae and more details.

### 3.1. Morphological Findings

Figures 1, 2, 3, 4, 5, 6, 7, 8, 9 show the morphological characteristics of the cervical vertebrae in the red fox using two‐dimensional (2D) and 3D CT scan images. Like other carnivores, there are seven cervical vertebrae in the red fox that were evaluated morphologically in this study.

Figure 1Transverse 2D computed tomography image (bone window) of the atlas vertebra in red fox, sections A and B. The picture at the top right of this figure shows the section of transverse CT images.

**Figure:**
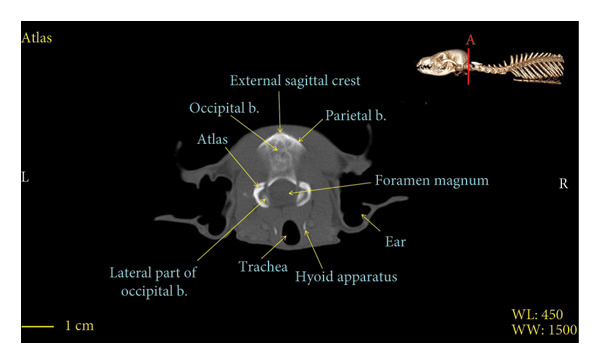
(a)

**Figure:**
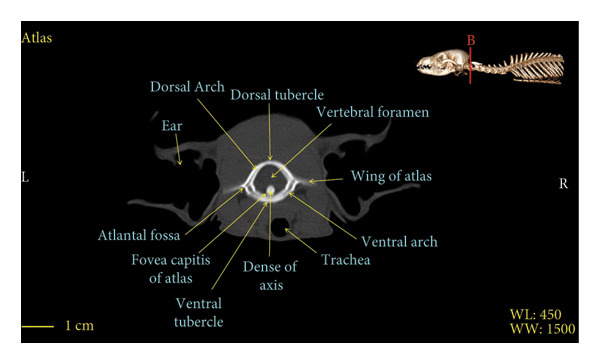
(b)

Figure 2Transverse 2D computed tomography image (bone window) of the axis vertebra in red fox, sections A and B. The picture at the top right of this figure shows the section of transverse CT images.

**Figure:**
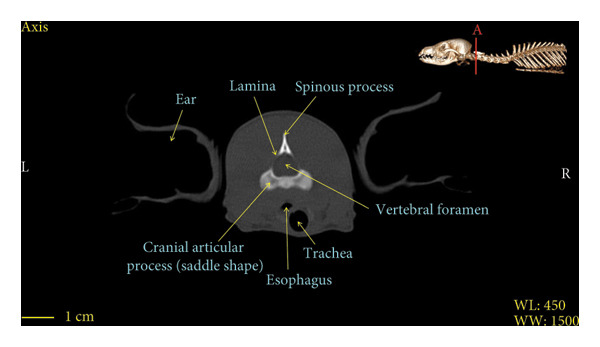
(a)

**Figure:**
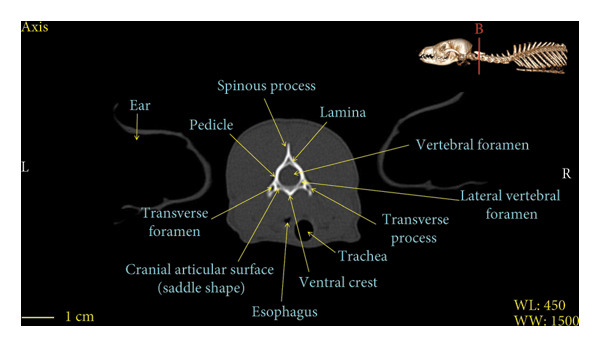
(b)

Figure 3Transverse 2D computed tomography image (bone window) of the C3 vertebra in red fox, sections A and B. The picture at the top right of this figure shows the section of transverse CT images.

**Figure:**
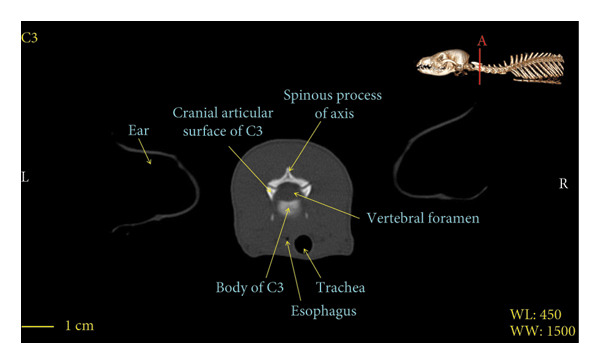
(a)

**Figure:**
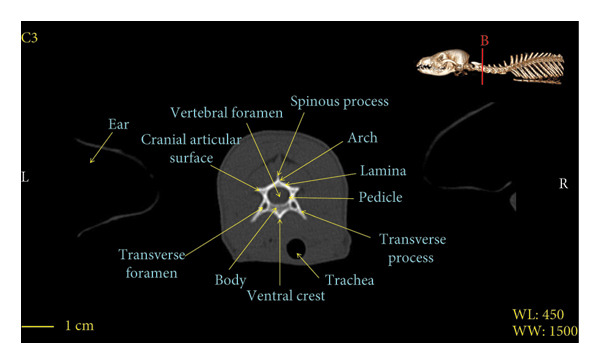
(b)

Figure 4Transverse 2D computed tomography image (bone window) of the C4 vertebra in red fox, sections A and B. The picture at the top right of this figure shows the section of transverse CT images.

**Figure:**
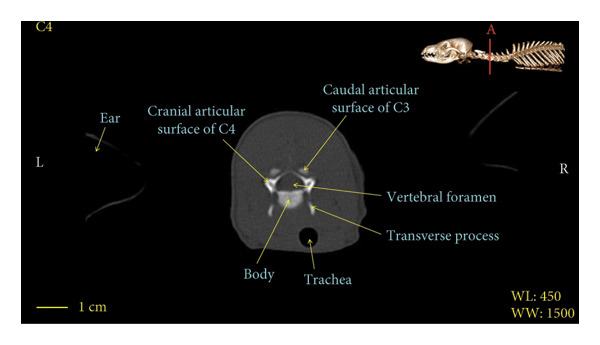
(a)

**Figure:**
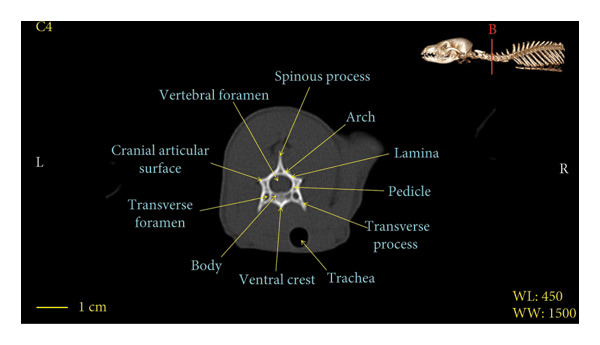
(b)

Figure 5Transverse 2D computed tomography image (bone window) of the C5 vertebra in red fox, sections A and B. The picture at the top right of this figure shows the section of transverse CT images.

**Figure:**
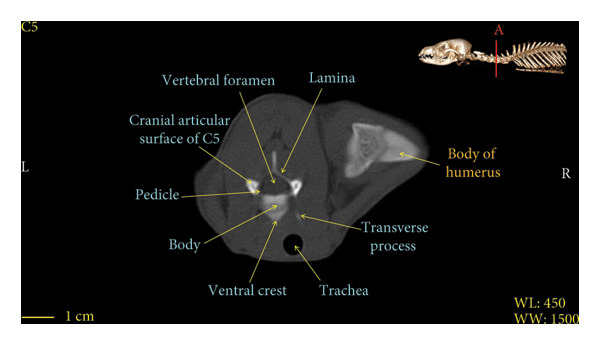
(a)

**Figure:**
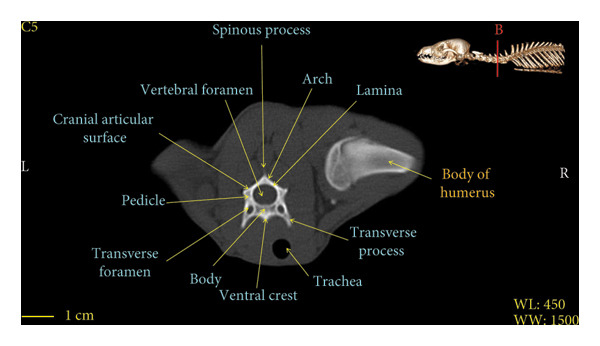
(b)

Figure 6Transverse 2D computed tomography image (bone window) of the C6 vertebra in red fox, sections A and B. The picture at the top right of this figure shows the section of transverse CT images.

**Figure:**
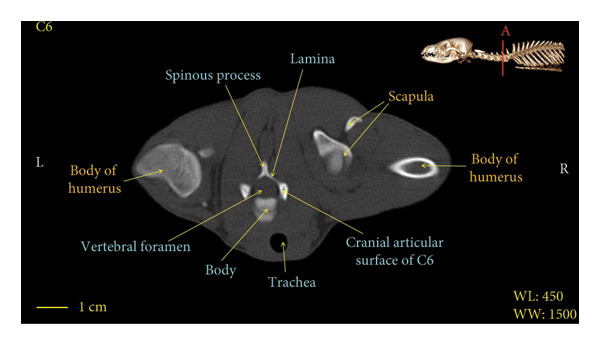
(a)

**Figure:**
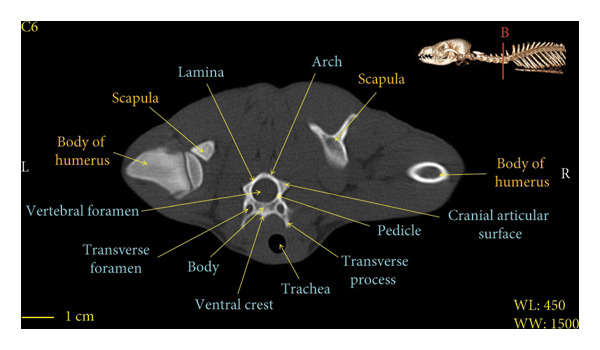
(b)

Figure 7Transverse 2D computed tomography image (bone window) of the C7 vertebra in red fox, sections A and B. The picture at the top right of this figure shows the section of transverse CT images.

**Figure:**
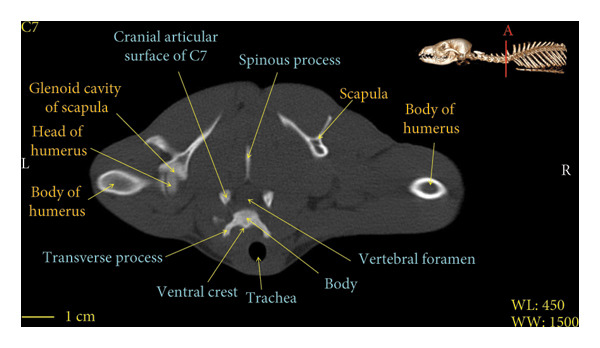
(a)

**Figure:**
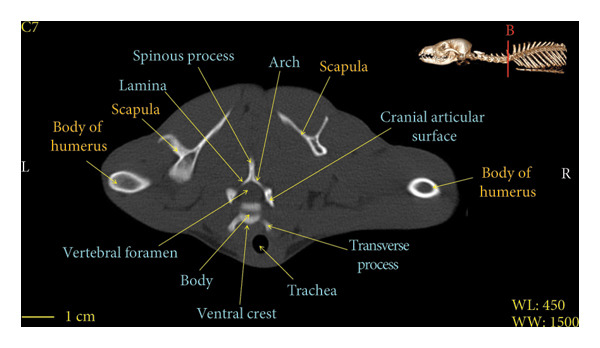
(b)

**Figure 8:**
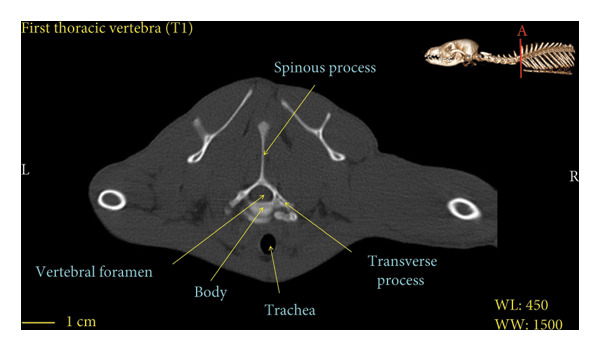
Transverse 2D computed tomography image (bone window) of the T1 vertebra in red fox. The picture at the top right of this figure shows the section of transverse CT images.

**Figure 9:**
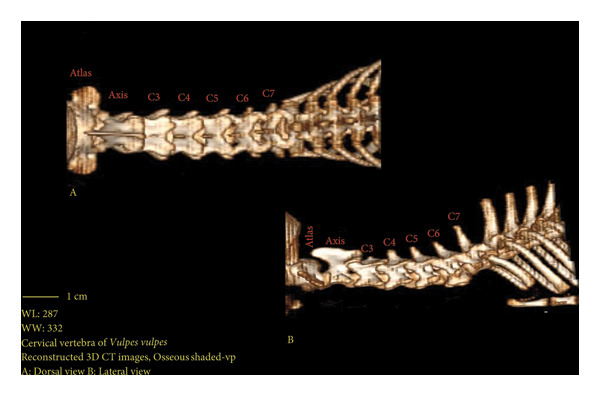
3D reconstruction computed tomography image of red fox cervical vertebrae. A. dorsal view, B. lateral view.

A hole‐shaped articular surface, called the cranial articular cavity, is seen in the front part of the atlas vertebra. The caudal articular process of this vertebra articulates with the same articular surface of the axis vertebra. At the front end of the axis (C2) vertebra, the cranial articular process has become a part called the dens, which is placed in the dental fossa of the atlas vertebra, as shown clearly in Figure 1, section B. The spinous process is large and robust, and the front part of the spinous process is stretched forward (Figures 1 and 9). Typical cervical vertebrae (C3, C4, and C5) have a short transverse process. The spinous process is also short in these vertebrae, but the transverse foramen is apparent (Figures 3, 4, 5). The cranial articular surface, transverse foramen, ventral crest, and transverse processes are well observed in the sixth cervical vertebra (Figure 6). In the seventh cervical vertebra (C7), the spinous process is slightly longer than in other vertebrae. Furthermore, the caudal part of the body on each side has an articulation surface to articulate with the ribs (Figures 7 and 8).

### 3.2. Morphometrical Observation

Morphometric studies have been conducted based on different parameters listed in Table 2. These items are measured in the cervical vertebrae of all five fox samples in “Radiant DICOM viewer” software, and Table 2 presents the results of its statistical analysis.

**Table 2:** Computed tomographic measurements of cervical vertebrae of red fox (mean ± SD in mm)^∗^.

Vertebra parameters	C1	C2	C3	C4	C5	C6	C7
VBH (mm)	—	6.64 ± 0.11^a^	6.34 ± 0.21^a^	6.77 ± 0.33^a^	6.59 ± 0.13^a^	6.10 ± 0.14^a^	6.34 ± 0.22^a^
SPH (mm)	—	6.10 ± 0.33^a^	3.17 ± 0.23^b^	5.86 ± 0.22^c^	6.83 ± 0.42^d^	7.96 ± 0.12^e^	11.3 ± 0.44^f^
TPL/WPL (mm)	9.59 ± 0.41^a^	6.04 ± 0.41^b^	7.81 ± 0.22^c^	7.44 ± 0.15^c^	7.45 ± 0.42^c^	7.88 ± 0.44^c^	7.76 ± 0.22^c^
TPW/WPW (mm)	38.7 ± 0.22^a^	17.13 ± 0.42^b^	23.23 ± 0.32^c^	22.52 ± 0.16^c^	23.72 ± 0.11^c^	23.31 ± 0.21^c^	22.91 ± 0.44^c^
VBL (mm)	—	13.14 ± 0.11^a^	13.26 ± 0.21^a^	13.48 ± 0.18^a^	13.32 ± 0.42^a^	13.52 ± 0.32^a^	13.31 ± 0.41^a^

^∗^The different letters in each column represent significant difference between vertebrae (*n* = 5, *p* < 0/05).

The difference in the VBH measurements of the vertebrae was not statistically significant (*p* > 0.05). On this basis, VBH did not change from the beginning to the end of the neck site. The difference in the SPH measurement of each vertebra with the previous vertebra was statistically significant (*p* < 0.05). On this basis, the SPH of the third vertebra was lower than that of the second vertebra, which then increased in each vertebra compared to the previous one.

The difference in the TPL measure of the first and second vertebrae was statistically significant (*p* < 0.05). The TPL of the first vertebra (WPL) was higher than other cervical vertebrae. The difference in the TPL measure of the third and second vertebrae was statistically significant (*p* < 0.05). The difference in the TPL measure of the vertebrae was not significant from the third vertebra to the last cervical vertebra (*p* > 0.05). On this basis, TPL was higher in the first vertebra than in others, decreased in the second vertebra, increased in the third vertebra, and remained constant.

The difference in the TPW measure of the first and second vertebrae was statistically significant (*p* < 0.05). The TPW of the first vertebra (WPW) was higher than other cervical vertebrae. The difference in the TPW measure of the third and second vertebrae was statistically significant (*p* < 0.05). The TPW measure of the vertebrae was insignificant from the third vertebra to the last cervical vertebrae (*p* > 0.05). Therefore, TPW was higher in the first vertebra than in others, decreased in the second vertebra, increased in the third vertebra, and remained constant.

The difference in the VBL measures of vertebrae was not statistically significant (*p* > 0.05). On this basis, VBL did not change from the beginning to the end of the neck site.

## 4. Discussion

There are seven cervical vertebrae in domestic animals, and the difference in neck length in different animals does not affect the number of vertebrae, while the measures of vertebrae are various in different animals [3]. Anatomical features of the seven cervical vertebrae in red foxes were investigated using the CT scan method in this study. This study, as the first research on cervical vertebrae in immature red fox using a CT scan to investigate its anatomical structures, can be used as a reference for other relevant studies, interpretation of injuries to vertebral columns, and diagnosis of pathological complications related to the neck. It can also help us to know more about different species of carnivores and the progress of taxonomy. Anatomical studies of the skeletal structures of an animal using modern diagnostic imaging techniques, such as a CT scan, are the basis of many new scientific studies.

In this study, the axis vertebra, similar to that of the White New Zealand Rabbit, features a dens that appears rod‐shaped and rounded [10]. In the red fox, the cervical vertebrae from C2 to C6 exhibit transverse foramina, comparable to those observed in the White New Zealand Rabbit. In contrast, the seventh cervical vertebra of the White New Zealand Rabbit presents a distinct transverse foramen, whereas in the red fox—as well as in species, such as dogs, sheep, and horses—the absence of this foramen is a characteristic feature of C7. Moreover, in the cervical vertebrae of the red fox, the spinous processes are oriented anteriorly, resembling the condition in the White New Zealand Rabbit [10, 11].

The examination of VBH in cervical vertebrae indicated that the measure of VBH was almost constant in cervical vertebrae, and they did not differ from each other. The comparison of VBH in cervical vertebrae with thoracic vertebrae in White New Zealand Rabbits and the insignificance of VBH in cervical and thoracic vertebrae showed almost equal rates of this parameter in cervical and thoracic vertebrae of White New Zealand Rabbits [12]. The difference in VBH measures of the vertebrae was not statistically significant in the study of VBH in red fox cervical vertebrae in this study, and thus, like White New Zealand Rabbits, this parameter did not change from the beginning to the end of the neck. Unlike White New Zealand Rabbits and red foxes, this difference was statistically significant in the specimens of Asian elephants, cattle, and horses. In the Asian elephants, this value increased in the third vertebra compared to the second vertebra, decreased in the fourth vertebra compared to the third, increased in the fifth vertebra and decreased again in the sixth vertebra, and finally remained constant in the sixth and seventh vertebrae. In cattle, this parameter was stable in the second and third vertebrae, increased in the fourth vertebra, decreased in the fifth vertebra compared to the fourth vertebra, and remained constant until the end of the seventh neck vertebra. In horses, this value increased in the third vertebra compared to the second vertebra, decreased in each vertebra until the sixth vertebra compared to its previous vertebra, and increased again in the seventh vertebra [10–13]. Consistent with these findings, a separate study on sheep reported a gradual reduction of this parameter along the craniocaudal axis [14].

The study of SPH in the cervical vertebrae of White New Zealand Rabbits, considering the nonsignificance of the SPH difference of the second to seventh cervical vertebrae, indicated that the SPH measures were almost constant and did not differ from each other [10]. Still, in red foxes, the SPH measure difference of each vertebra with the previous vertebra was statistically significant. It is worth noting that the SPH of the third vertebra was lower than the second vertebra, which then increased in each vertebra compared to the previous vertebra. This difference was statistically significant in specimens of Asian elephants, cattle, and horses similar to the red fox. Therefore, this parameter decreased in the third vertebra compared to the second in Asian elephants and cattle. It was stable in the third and fourth vertebrae and then increased until the seventh vertebra in each vertebra compared to the previous vertebra. In horses, this parameter decreased in the third vertebra compared to the second, then increased in the fourth vertebra and decreased again in the fifth vertebra, and then increased in the sixth and seventh vertebrae in each vertebra compared to the previous vertebra. In the Asian elephant and cattle specimens, the highest value was related to the seventh vertebra, and the lowest value was associated with the third and fourth vertebrae. In horses, the highest value was seen in the second vertebra, and the lowest was observed in the third vertebra [13].

In examining TPL in the cervical vertebrae of White New Zealand Rabbits, considering the significant difference between the TPL of the first cervical vertebra and other cervical vertebrae, the TPL was longer in the first cervical vertebra than in cervical vertebrae. The length of the wing of the first cervical vertebra (atlas) was longer than the length of the transverse process of other cervical vertebrae. The measure of this parameter was almost constant and did not change in other cervical vertebrae of rabbits [10]. In red foxes, the difference in TPL measures of the first and second vertebrae was statistically significant. Furthermore, the TPL of the first vertebra was higher than the other cervical vertebrae, and it can be concluded that TPL was higher in the first vertebra than the others, decreased in the second vertebra, increased in the third vertebra, and then remained constant. According to the comparison between the TPL of cervical and thoracic vertebrae in White New Zealand Rabbits, the length of the transverse process in the atlas vertebra, which was the wing of the atlas (WPL), was greater than the length of this process in the thoracic vertebrae [12].

The examination of cervical vertebrae TPW in red fox indicated that TPW was higher in the first vertebra than others, decreased in the second vertebra, increased in the third vertebra, and remained constant. In White New Zealand Rabbits, the width of the transverse process (wings in the case of this vertebra) was greater in this vertebra than in other vertebrae. According to the results of statistical research conducted by Shateri Amiri, it was observed that the width of the transverse process in the second, third, and fourth vertebrae of the neck was less than the first vertebra, but they were almost equal to each other. Furthermore, it increased again in the last three cervical vertebrae but was almost equal in the three vertebrae [10]. Comparing the TPW of cervical vertebrae with thoracic vertebrae of White New Zealand Rabbits indicated that this parameter was different and smaller in thoracic vertebrae than in cervical vertebrae. It was also concluded that the measures of TPW in the atlas and thoracic vertebrae were not equal, and they were larger in the atlas vertebra [12]. This difference was statistically significant in the specimens of Asian elephants, cattle, and horses, so this value increased from the beginning to the end in each vertebra in Asian elephants compared to the previous vertebra. In cattle, this value was constant from the second to the fifth vertebra, decreased in the sixth vertebra compared to the previous vertebra, and increased in the seventh vertebra. In horses, this parameter increased in the third vertebra compared to the second vertebra, remained constant in the fourth vertebra, decreased in the fifth vertebra, increased in the sixth vertebra, and remained constant in the sixth and seventh vertebrae [13].

The difference in the VBL value of the vertebrae was not statistically significant, neither in red foxes nor in White New Zealand Rabbits [12]. Therefore, VBL did not change from the beginning to the end of the neck site, but unlike White New Zealand Rabbits and red foxes, the difference was statistically significant in specimens of Asian elephants, cattle, and horses. In the Asian elephants, this value decreased from the second to the fourth vertebra, increased in the fifth vertebra compared to the fourth vertebra, remained constant in the sixth vertebra, and increased in the seventh vertebra compared to the previous vertebra. Therefore, the highest maximum value was recorded in the second vertebra, and the lowest value was noted in the fourth vertebra. In cattle, this value decreased in the third vertebra compared to the second vertebra, remained constant until the fifth vertebra, decreased in the sixth vertebra compared to the previous vertebra, and increased in the seventh vertebra compared to the sixth. The highest value was observed in the second vertebra, and the lowest value was in the sixth vertebra in cattle. In horses, this parameter decreased from the second to the fourth vertebra compared to the previous vertebra, remained constant until the sixth vertebra, and again decreased in the seventh vertebra compared to the sixth vertebra. Therefore, the highest value was seen in the second vertebra and the lowest in the seventh vertebra [13].

## 5. Conclusion

In summary, the application of CT imaging in this study has provided valuable insights into the cervical vertebrae of the red fox. The acquisition of high‐resolution 2D and 3D images allowed for precise assessment of vertebral morphology, revealing subtle transitional features and notable structural variations. Although the limited sample size presented certain constraints, the methodology effectively balanced the need for rapid imaging with the preservation of specimen integrity, while still achieving comprehensive anatomical detail. Collectively, these findings not only corroborate previous anatomical knowledge but also offer a solid foundation for future comparative studies in vertebral morphology.

## Ethics Statement

This study was approved by the Administration Committee of the Basic Sciences Department, Lorestan University. All of the experimental procedures involving animals were conducted in accordance with the Institutional Animal Care guidelines of Lorestan University with the verified edictal code of ethics committee in animal research (LU. ECRA.2019.22).

## Conflicts of Interest

The authors declare no conflicts of interest.

## Author Contributions

Yasin Valizadeh: funding acquisition and writing–review and editing. Mohsen Abbasi: funding acquisition, validation, and writing–review and editing. Omid Zehtabvar: conceptualization, data curation, software, and writing–original draft preparation. Amir Zakian: methodology, project administration, supervision, and writing–review and editing. Ali Reza Vajhi: formal analysis, resources, software, and writing–original draft preparation. Ferdos Fekri: supervision, writing–review and editing, and data curation.

## Funding

No funding was received for this research.

## Data Availability

The data that support the findings of this study are available from the corresponding author upon reasonable request.
